# Tobacco and Health Disparities

**DOI:** 10.1155/2015/570173

**Published:** 2015-07-27

**Authors:** Kamran Siddiqi, Mohammed Jawad, Nasir Mushtaq, Shehzad Ali, Javaid Ahmed Khan

**Affiliations:** ^1^Department of Health Sciences, University of York, York YO10 5DD, UK; ^2^Department of Primary Care and Public Health, Imperial College London, London W6 8RP, UK; ^3^Academic Unit of Primary Care and Population Sciences, University of Southampton, Southampton SO16 6YD, UK; ^4^Department of Biostatistics and Epidemiology, University of Oklahoma Health Sciences Center, 4502 E. 41st Street, SAC 1G06, Tulsa, OK 74135, USA; ^5^Section of Pulmonary and Critical Care Medicine, Department of Medicine, The Aga Khan University, Karachi 74800, Pakistan

Tobacco use is a major threat to public health and if current consumption patterns remain unchanged, it will result in one billion deaths in 21st century [1]. A combination of public health efforts in recent years has resulted in a general decline in the prevalence of tobacco use worldwide; however, the total number of smokers has increased due to population growth [2]. Furthermore, the most poor, marginal, and vulnerable sections of the society have not benefitted from such efforts and hence tobacco consumption remains high in these groups leading to devastating consequences and rising health inequalities [3, 4]. These include those with low socioeconomic status, homeless people, indigenous and minority ethnic groups, and patients with debilitating conditions such as tuberculosis, HIV, and mental disorders. This is particularly devastating as their disadvantage increases their likelihood of consuming tobacco as a “coping strategy” and, subsequently, their tobacco use increases their disadvantage through poor health, less money for essentials, and economic burden [1]. In order to achieve further decline in global tobacco consumption, tobacco control communities need to focus their efforts on reducing tobacco-related health disparities. There are a number of potential barriers to such efforts. Firstly, we understand very little about how exposure to disadvantaged circumstances shapes smoking careers throughout the life course [5]. Secondly, measures such as socioeconomic status are often not included in the evaluation of tobacco control interventions. Thirdly, tobacco control interventions are often not tailored to the particular needs of disadvantaged populations. Finally, tobacco control policy is generally not linked to policies to tackle social determinants of health [6]. It is, therefore, not a surprise that, apart from taxation measures, tobacco control interventions appear to have very little effect on reducing health inequalities [7]. In this special issue, we have included eleven research articles that help to expand our understanding of social disparities in tobacco use and highlight the need for progressive approaches to tackle these.

Three studies by M. Lund, N. J. Grills et al., and F. Janssen and F. van Poppel remind us of the role of education, occupation, and gender in determining the course of tobacco epidemic. In a study of 1,200 Norwegian smokers that used successive cross-sectional data, M. Lund demonstrated a strong association between low levels of education and high levels of cigarette consumption, dependence, and lack of intention to quit. In another large study of tobacco prevalence and attitudes from North India, where nearly 70% of men are tobacco users, N. J. Grills et al. identified a range of educational and occupational disparities in tobacco use. Applying a historical perspective, F. Janssen and F. van Poppel examined gender differences in smoking adoption patterns in Netherlands and found that these differences played a major role in differences in life expectancy and smoking related cancer mortality between men and women.

Two other studies by K. A. Vickerman et al. and R. Hiscock et al. examined the distribution and determinants of low cessation rates in tobacco quitline programme in three USA states and in English Stop Smoking Services, respectively. In the USA-based study, K. A. Vickerman et al. followed up 3,262 clients for a period of seven months and found that the self-reported quit rates among those with one or more than one mental health condition were lower than those without. Authors concluded that, for those with mental health conditions, cessation programmes such as quitline need to be tailored accordingly. In the UK-based study, R. Hiscock et al. identified some important factors, which contribute to lower cessation rates among smokers who belong to lower socioeconomic status. Material factors, such as housing tenure, along with social factors and use of cessation medication were identified as significantly associated with smoking abstinence among this group.

In a series of three studies, A. Singh et al., M. Jawad et al., and M. Jawad et al. examined noncigarette forms of tobacco, a neglected but important topic from the perspective of vulnerable populations. In a secondary analysis of Global Adult Tobacco Survey (GATS) 2009-10 data, A. Singh et al. demonstrated that in India social gradient for tobacco use changes with the type of tobacco products where cigarette smoking is common among wealthier individuals while bidi smoking and smokeless tobacco are common among impoverished and less educated. The other two studies focused on waterpipe smoking, a traditional form of tobacco smoking in Middle East and South Asia but a relatively modern trend among young people in Europe and USA. In a qualitative study, M. Jawad et al. assessed the impact of health warning labels on waterpipe. The findings highlights that noncigarette forms of tobacco may not be as sensitive to existing tobacco control legislation as cigarettes, and in attempt to address disparities we may need to adapt our interventions accordingly. In the other paper by M. Jawad et al., the longitudinal analysis of a simple social media campaign gives us insight into how disparities in intervention effects can be unique to different social media platforms. The description of a low-resource social media campaign may be a valuable tool for those wishing to embark on mass media campaigns for further tobacco control interventions.

Two of the studies included here by A. J. Saari et al. and A. H. Al-Zalabani et al. remind us of the importance of adolescence in establishing tobacco-related norms. In a longitudinal study in Finland, A. J. Saari et al. showed the predictive effect of low self-esteem during adolescence on subsequent smoking behaviours during adulthood. In another school-based survey in Saudi Arabia, A. H. Al-Zalabani et al. demonstrated a considerable high prevalence of second-hand smoke exposure among adolescents, which was strongly associated with the smoking behaviours of their parents, peers, and other family members.

Finally, in a systematic review examining the epidemiology of tobacco use among khat users, S. Kassim et al. emphasize the high prevalence of tobacco use among people who chew khat, a socially acceptable mild amphetamine popular in parts of the Middle East and East Africa. Not only does this remind us that risk behaviours come in tandem, but also the review provides interesting hypotheses about the synergism that khat plays in the dependence profile of tobacco users.

*Kamran Siddiqi* *Mohammed Jawad* *Nasir Mushtaq* *Shehzad Ali* *Javaid Ahmed Khan*
